# Regeneration, genetic transformation, and gene editing in *Vaccinium* species: current progress and future directions

**DOI:** 10.1093/hr/uhag217

**Published:** 2026-06-03

**Authors:** Anandi Karn, Felix Enciso-Rodriguez, Yaiphabi Kumam, Masafumi Omori, Kevin Begcy, Patricio Munoz

**Affiliations:** Blueberry Breeding Program, Horticulture Sciences Department, University of Florida, Gainesville, FL 32611, USA; Blueberry Breeding Program, Horticulture Sciences Department, University of Florida, Gainesville, FL 32611, USA; Blueberry Breeding Program, Horticulture Sciences Department, University of Florida, Gainesville, FL 32611, USA; Blueberry Breeding Program, Horticulture Sciences Department, University of Florida, Gainesville, FL 32611, USA; Laboratory of Pomology, Graduate School of Agriculture, Kyoto University, Kyoto, Japan; Microbiology and Cell Science Department, University of Florida, Gainesville, FL 32611, USA; Blueberry Breeding Program, Horticulture Sciences Department, University of Florida, Gainesville, FL 32611, USA; Plant Breeding Graduate Program, University of Florida, Gainesville, FL 32611, USA

## Abstract

*Vaccinium* is one of the most economically significant genera in the Ericaceae family, with breeding efforts substantially improving yield, fruit quality, and adaptation, contributing to the widespread consumption of this berry-bearing clade. Crops within this genus are valued for their high nutritional content, including antioxidants, dietary fiber, and vitamins, as well as their culinary versatility. Health benefits, such as anti-inflammatory activity, combined with advances in breeding and cultivation that enhance flavor and yield, have further boosted consumer demand. However, the application of traditional breeding methods in *Vaccinium* remains challenging, mostly due to their long juvenile period, polyploidy, and the complex genomes of both cultivated and wild plants. To overcome these constraints, new breeding techniques (NBTs), such as gene editing, offer tremendous potential for developing improved varieties with traits that are difficult to achieve through traditional methods. Over the past 30 years, significant advancements in *Vaccinium* biotechnology have been made, particularly in the field of transgenesis. These advancements, including the ability to introduce foreign genes and edit genomes, hold great promise for overcoming the challenges posed by traditional breeding techniques. Despite these developments, the implementation of NBTs in *Vaccinium* remains at an earlier stage compared with other horticultural crops, primarily due to: (i) recalcitrancy to stable genetic transformation and (ii) low editing efficiencies. In this review, we provide a detailed overview of advancements in regeneration and genetic transformation in *Vaccinium* species, along with a perspective on the major constraints and future strategies to improve *Vaccinium* through biotechnological methods.

## Introduction

*Vaccinium* is a common and widespread genus from the Ericaceae family, consisting of >450 species [1]. This taxon is divided into 29 sections, containing species with different ploidy levels (2×–6×) distributed widely across the globe, excepting Australia and Antarctica [1, 2]. *Vaccinium* includes various nutritionally significant and commercially valuable berry crops, such as blueberry (*Vaccinium corymbosum* L.), cranberry (*V*. *macrocarpon* L.), bilberry (*V*. *myrtillus* L.), lingonberry (*V. vitis*-*idaea* L.), and ohelo berry (*V. reticulatum* L.) [3]. Recently, global demand for these species has increased due to their flavor, antioxidant content, nutritional value, and other potential health benefits [4].

The implementation of effective breeding strategies is crucial for meeting the growing demand for these traits, as well as mitigating the negative impacts of biotic and abiotic stresses.

Although conventional breeding methods have been widely used for developing new *Vaccinium* cultivars, the presence of polyploidy and high heterozygosity often makes traditional breeding a time- and labor-intensive process [3]. Additionally, in cases such as blueberry, current cultivars are derived from interspecific crosses, which often leads to linkage drag, making it challenging to develop a desirable cultivar without inheriting undesirable traits [5].

Biotechnological approaches have provided alternative strategies to overcome these limitations in other crops. For instance, transgenesis has enabled the direct insertion of genes into desired plant cultivars, providing an efficient strategy for trait improvement and plant functional genomics [6]. Furthermore, the advancements of these molecular techniques have established the groundwork for new breeding techniques (NBTs), which include site-directed nucleases such as Clustered Regularly Interspaced Short Palindromic Repeats (CRISPR) systems [7]. These novel strategies enable precise modification of an intended genome through targeted DNA cleavage and repair pathways [8]. They can also accelerate crop improvement by significantly shortening the breeding cycle and enhancing selection efficiency [7]. Therefore, in the current scenario, NBTs can offer an alternative approach and could be integrated with conventional breeding methods to achieve targeted gene modification and accelerate the improvement of *Vaccinium* crops.

One way to achieve a successful implementation of NBTs in any crop is through an efficient and reproducible *in vitro* shoot regeneration and transformation system, as it provides the essential foundation for gene delivery and the development of transgenic plants [9]. An ideal transformation system should provide high regeneration rates, genetic stability, and reproducibility across diverse genotypes, thus offering a reliable basis for advanced genetic manipulation [10]. This requires fine-tuning the balance of plant growth regulators (PGRs), along with explant choice, growth conditions, and basal medium [11]. Due to the perennial and woody nature of *Vaccinium* crops, plant regeneration can be particularly challenging, directly affecting the implementation of NBTs. This is further hindered by genotype-dependent shoot regeneration, low efficiency, and recalcitrance to transformation and gene editing observed in this genus [12–14].

In this review, we provide a comprehensive analysis of the current literature on shoot regeneration and genetic transformation in *Vaccinium* species. It first outlines the major factors influencing regeneration efficiency, including PGRs, explant types, and environmental conditions. Then, we summarize current advances in *Vaccinium* transformation and gene editing, emphasizing key targeted traits. Finally, we identify the persistent bottlenecks limiting regeneration and transformation efficiency and discussed emerging methodologies with potential to accelerate genetic improvement in *Vaccinium*.

## An overview of past and current research on shoot regeneration in *Vaccinium*

Plant regeneration relies on ‘totipotency,’ the ability of plant cells to develop whole plants under *in vitro* conditions. This process occurs mainly through somatic embryogenesis or *de novo* organogenesis, either directly from explants or indirectly via callus formation, with nonmeristematic tissues such as leaves, cotyledons, and hypocotyls often serving as explant sources [15, 16]. In *Vaccinium*, early research efforts focused on creating effective protocols for shoot regeneration. A key milestone was the first successful *in vitro* regeneration of lowbush blueberry (*V*. *angustifolium*) using cotyledon and hypocotyl sections ([192]; Table 1). Since this pioneering work over four decades ago, research on regeneration in this genus has significantly increased (Fig. 1A).

**Table 1:** Reported regeneration studies in *Vaccinium*

Species (common name)	Cultivar/Genotype	Explant	Media composition			References
			Basal salt	Auxin (μM)	Cytokinin (μM)	
*V. corymbosum* (Highbush blueberry)	‘Albus’	Leaf	WPM	-	tZ (40.6)	[25]
	‘Arcadia’	Leaf	WPM	-	tZ (18.24)	[25]
	‘Aurora’	Leaf	MWPM	NAA (2.68)	TDZ (4.54)	[29]
			WPM	-	TDZ (5)	[70]
	‘Berkeley’	Leaf	WPM	-	2iP (10)	[18]
			AN	-	Z (9.12)	[28]
	‘Biloxi’	Leaf	WPM	-	ZR (11.4)	[26]
			Leaf and stem	-	Z (4.56)	[24]
	‘Bluecrop’	Leaf	½ MS	-	2iP (25)	[164]
			PT1- N6 + LS PT2 – MWPM	-	TDZ (1)	[42]
			MWPM	-	2iP (24.6) + Z (9.1)	[29]
			AN	-	Z (9.12)	[28]
		Leaf and stem	WPM	-	Z (13.68) + 2iP (9.84)	[20]
			Leaf	IBA (4.92)	Z (9.12)	[14]
	‘Bluegold’	Leaf and stem	WPM	-	Z (13.68) + 2iP (9.84)	[20]
	‘Bluehaven’	Leaf	WPM	-	2iP (15)	[18]
	‘Bluejay’	Leaf	WPM	IBA (2.46)	Z (9.12)	[13]
			Leaf and stem	-	Z (13.68) + 2iP (9.84)	[20]
	‘Blue Muffin’ (‘BM’)	Leaf and axillary bud	WPM	-	Z (10.03)	[84]
		Leaf	MW	-	TDZ (4.54)	[12]
	‘Blueray’	Leaf	AN	-	Z (9.12)	[28]
	‘Brigitta’	Leaf	MWPM	-	2iP (24.6 ) + Z (9.12)	[29]
			WPM	IBA (4.92)	Z (9)	[14]
			MW	-	TDZ (4.54)	[12]
	‘Brigitta Blue’	Leaf	WPM	NAA (1.07)	Z (18.24)	[23]
			Petiole and stem	WPM	IAA (1.14)	Z (18.24)
	‘Chandler’	Leaf	WPM	IBA (4.92)	Z (9.12)	[14]
	‘Colossus’	Leaf	WPM	-	tZ (10.03)	[25]
	‘Darrow’	Leaf	AN	-	Z (9.12)	[28]
	‘Duke’	Leaf	MWPM	-	TDZ (1 or 5) and Z (20)	[19]
			MWPM	-	TDZ (1)	[29]
			AN	-	Z (9.12)	[28]
			WPM	-	CPPU (4.03)	[68]
				-	Z (9.12)	[166]
				-	Z (9.12)	[167]
				IBA (4.92)	Z (9.12)	[14]
		Leaf and stem	WPM	-	Z (2.28)	[24]
				-	2iP (9.84) + BAP (4.44)	[168]
	‘Emerald’	Leaf	WPM	-	TDZ (4.54 or 9.08), Z (18.2), ZR (11.4)	[26]
					tZ (18.2) + 2iP (2.46)	[64]
				IBA (2.46)	Z (13.6)	[114]
	‘Elliott’	Leaf	MWPM	-	2iP (24.6 ) + Z (9.12)	[29]
	FL14-242	Leaf	WPM	-	TDZ(1) and tZ (10.03)	[25]
	‘Georgiagem’	Leaf	MWPM	-	Z (20)	[19]
			WPM	-	CPPU (12.09)	[68]
	‘Gulfcoast’	Leaf	WPM	IBA (4.92)	Z (9.12)	[14]
	‘JE’	Leaf	WPM	-	CPPU (8.06) + 2-iP (0.98)	[11]
			Stem	MS	IAA (1.14)	CPPU (0.4)
	‘Jersey’	Leaf	MWPM	-	Z (20)	[19]
	‘Jewel’	Leaf	WPM	NAA (2.69)	TDZ (4.54)	[26]
				-	tZ (18.2) + 2iP (4.92)	[64]
	‘Jubilee’	Leaf	WPM	NAA (2.69)	TDZ (4.54), ZR (5.7 or 11.4)	[26]
	‘Keecrisp’	Leaf	WPM	-	tZ(40.06)	[25]
	‘Lanmei No. 1’	Leaf	WPM	-	TDZ (0.23)	[169]
	‘Legacy’	Leaf	MWPM	-	2iP (24.6 ) + Z (9.12)	[29]
			WPM	IBA (4.92)	Z (9.12)	[14]
			MW	-	TDZ (4.54)	[12]
			WPM	-	tZ (18.2) + 2iP (2.46)	[64]
				NAA (2.69)	TDZ (9.08), Later Z (13.68)	[88]
			Leaf and stem	-	Z (4.56)	[24]
	‘Misty’	Leaf	WPM	-	Z (20)	[170]
	‘M5’	Leaf	WPM	-	ZR (11.4)	[171]
	‘NC5288’	Leaf	WPM	-	tZ (18.2) + 2iP (4.92)	[64]
	‘Northblue’	Stem	Z-2	-	Z (25)	[22]
	‘North sky blue’	Leaf	WPM	-	DMAAP (14.76)	[21]
	‘North Country’	Leaf	WPM	-	DMAAP (14.76)	[21]
	‘O’Neal’	Stem	OM	NAA (10.74)	Z (9.12) + K (0.23)	[49]
		Leaf	WPM	IBA (4.92)	Z (9.12)	[14]
				NAA (2.68)	TDZ (4.42)	[84]
			MW	-	TDZ (4.54)	[12]
	‘Optimus’	Leaf	WPM	-	tZ (40.06)	[25]
	‘Ozarkblue’	Leaf	WPM	-	Z (20)	[65]
	‘Patriot’	Leaf and stem	WPM	-	Z (13.68) + 2ip (9.84)	[20]
	‘Pinnacle’	Leaf	WPM	-	tZ (18.2) + 2iP (4.92)	[64]
	‘Rowan’	Leaf	WPM	-	tZ (18.2) + 2iP (2.46)	[64]
	‘Spartan’	Leaf and stem	WPM	-	Z (13.68) + 2iP (9.84)	[20]
	‘Sierra’	Leaf	MWPM	-	TDZ (1 or 5) and Z (20)	[19]
	‘Sharpblue’	Leaf	WPM	IBA (4.92)	Z (9.12)	[14]
	‘Sunrise’	Leaf	MWPM	-	ZR (20)	[66]
					-	[165]
			WPM	-	CPPU (4.03)	[68]
	‘Sunshine blue’	Leaf	WPM	IBA (4.92)	Z (9.12)	[14]
		Leaf, stem and callus		IBA (2.46)	Z (9.12)	[13]
	‘Toro’	Leaf and stem	WPM	-	Z (13.68) + 2iP (9.84)	[20]
			Leaf and stem	-	Z (9.12)	[23]
			Petiole	IAA (1.14)	Z (9.12)	[23]
*V. angustifolium* (Lowbush blueberry)	‘Blomidon’	Leaf	WPM	IBA (4.92)	Z (9.12)	[14]
	NA	Cotyledon and hypocotyl	AN	IAA (29.6)	2ip (110)	[192]
	NA	Pericarp callus	NM	L-Tryp (489.7)	K (0.46)	[172]
	‘PB1 clone’	Leaf	BM	-	TDZ (2.3 - 4.5)	[75]
				-	TDZ (1.2 - 2.3)	[173]
	‘QB1 clone’	Leaf	BM	-	TDZ (2.3 - 4.5)	[75]
				-	TDZ (1.2 - 2.3)	[173]
	‘QB2 clone’	Leaf	BM	-	TDZ (2.3 - 4.5)	[75]
				-	TDZ (1.2 - 2.3)	[173]
*V. virgatum* or *V. ashei* (rabbiteye blueberry)	‘Balduin’	Leaf	WPM	NAA (0.1)	2iP (73.5)	[174]
	‘Baldwin’	Leaf	WPM	IBA (4.92)	Z (9.12)	[14]
	‘Bluebell’	Leaf	WPM	NAA (0.1)	2iP (73.5)	[174]
	‘Brightwell’	Leaf	WPM	IBA (4.92)	Z (9.12)	[14]
	‘Choice’	Leaf	WPM	NAA (0.1)	2iP (73.5)	[174]
	‘Dellite’	Stem	WPM	NAA (0.1)	2iP (73.5)	[174]
			Leaf	-	TDZ (0.5)	[44]
	‘Gardenblue’	Leaf	WPM	IBA (4.92)	Z (9.12)	[14]
		Leaf	WPM	NAA (0.1)	2iP (73.5)	[174]
	‘Premier’	Leaf	WPM	IBA (4.92)	Z (9.12)	[14]
				NAA (0.1)	2iP (73.5)	[174]
	‘Pink Lemonade’^a^	Leaf	WPM	-	Z (9.12)	[13]
				IBA (4.92)	Z (9.12)	[14]
	‘Powderblue’	Leaf	WPM	NAA (0.1)	2iP (73.5)	[174]
*V. macrocarpon* (cranberry)	‘Ben Lear’	Leaf	AN + MS	-	TDZ (10) + 2iP (5)	[63]
		Stem	BM	-	2iP (12.3 - 24.6)	[175]
	‘Bergman’	Leaf	AN + MS	NAA (1)	TDZ (10)	[27]
	‘Early Black’	Leaf	AN + MS	-	TDZ (10) + 2iP (5)	[63]
	‘Franklin’	Leaf	AN + MS	NAA (1)	TDZ (10)	[27]
	‘Pilgrim’	Leaf	AN + MS	-	TDZ (10) + 2iP (5)	[63]
		Stem	BM	-	2iP (12.3 - 24.6)	[175]
	‘Stevens’	Leaf	AN + MS	-	TDZ (10) + 2iP (5)	[63]
	‘No. 35’	Leaf	AN + MS	-	TDZ (10) + 2iP (5)	[63]
*V. vitis-idaea* (lingonberry)	NA	Leaf	BM-D	-	Z (9.1), TDZ (1.8)	[176]
	‘ECL1’	Leaf	BM-D	-	Z (10)	[41]
	‘Erntedank’	Leaf	BM	-	TDZ (5)	[71]
			BM	-	Z (5)	[177]
			BM	-	Z (4)	[72]
	‘Ida’	Hypocotyl	MWPM	-	TDZ (5)	[178]
	‘Koralle’	Leaf	AN	-	Z (3.42)	[28]
	‘Red Pearl’	Leaf	AN	-	Z (3.42)	[28]
			WPM	NAA (1)	Z (20)	[65]
	‘Regal’	Leaf	BM	-	Z (10 - 20)	[41]
			BM	-	Z (5)	[177]
		‘Splendor’	BM	-	Z (5)	[41]
*V. myrtillus* (billberry)	NA	Leaf and hypocotyl	WPM	-	TDZ (0.9 - 2.7)	[76]
	NA	Stem	WPM	IBA (0.492)	Z (4.56)	[179]
	NA	Leaf and stem	WPM	IBA (2.46)	Z (9.12)	[87]
*V. deliciosum* (cascade huckleberry)	‘VADE 004A’	Leaf and stem	BM-D	-	Z (9.1 – 13.7)	[180]
*V. membranaceum* (mountain huckleberry)	‘VAME 031C’	Leaf and stem	BM-D	-	Z (9.1 – 13.7)	[180]
*V. ovalifolium* (oval-leaf bilberry)	‘VAOF 032D’	Leaf and stem	BM-D	-	Z (9.1 – 13.7)	[180]
*V. reticulatum* (ohelo berry or Hawaiian blueberry)	‘Red Button’	Leaf	WPM	IBA (4.92)	Z (9.12)	[14]
	‘Ohelo’	Leaf	WPM	-	TDZ (0.9 - 2.7)	[76]
*V. uliginosum* L. (alpine blueberry)	NA	Stem	WPM	IBA (0.492)	Z (4.56)	[179]
*V. floribundum* (Mortiño or Andean blueberry)	NA	Axillary buds	MWPM	NAA (0.53)	2ip (24.6)	[181]
	NA	Leaf	WPM	-	TDZ (6.81)	[182]
*V. arctostaphylos* (Caucasian whortleberry)	NA	Leaf	AN	IAA (1.148)	Z (4.56 and 13.68)	[183]
	NA	Stem	WPM	IBA (0.492)	Z (4.56)	[179]
*V. corymbosum* x *V. elliottii*	NA	Leaf	MKM	-	2ip (24.6)	[184]
*V. corymbosum* x *V. angustufolium* (half-highbush blueberry)	‘Chippewa’	Leaf	BM	-	TDZ (9.08)	[185]
	‘Northland’	Leaf	MWPM	-	Z (18.24)	[51]
	‘Northblue’	Leaf	BM	-	TDZ (9.08)	[185]
	‘Patriot’	Leaf	BM	-	TDZ (9.08)	[185]
	‘St. Cloud’	Leaf	BM	-	TDZ (9.08)	[185]
	‘Top Hat’	Leaf	WPM	IBA (1.23)	Z (9.12)	[13]

Abbreviations: WPM, woody plant medium; MWPM, modified woody plant medium; AN, Anderson medium; BM, basal medium; MBM, modified basal medium; MS, Murashige and Skoog medium; N6, Chu’s medium; LS, Linsmaier and Skoog medium; OM, olive medium; MKM, modified Knop’s medium; NM, Nitsch’s medium; Z-2, Zimmermann’s medium; PT1, Pre-treatment 1; PT2, Pre-treatment 2; MW, 1/2 WPM and 1/2 MS; Z, zeatin; ZR, zeatin riboside; tZ, trans zeatin; TDZ, thiadizuron; IAA, indole acetic acid; IBA, indole butyric acid; NAA, naphthalene acetic acid; 2iP, isopentenyladenine; DMAAP, 6-(γ,γ-dimethylallylamino)purine; CPPU, Forchlorfenuron; K, Kinetin; L-Tryp, Tryptophan.

a *V. virgatum* hybrid derivative.

**Figure 1:**
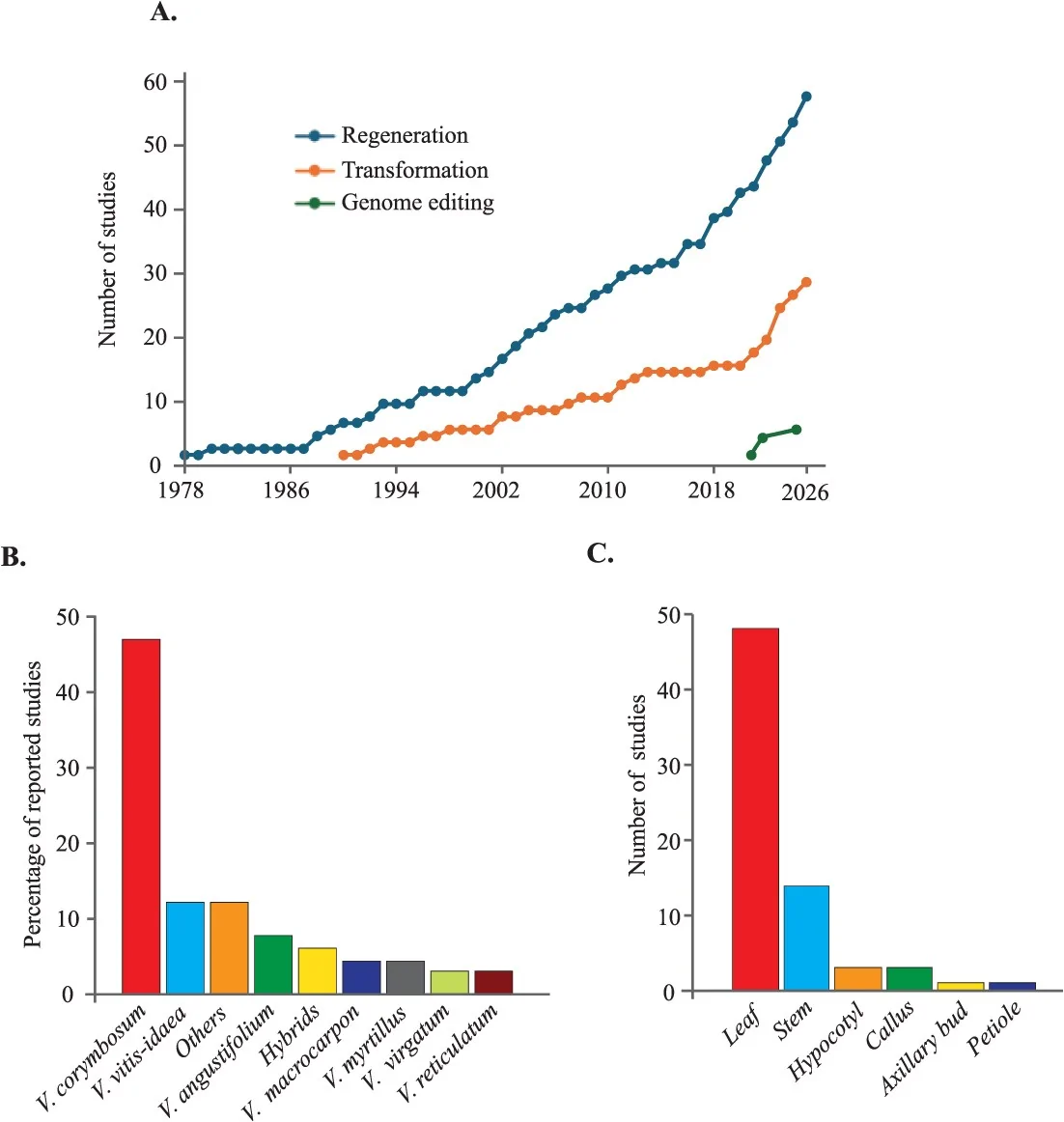
Trends and distribution of regeneration, transformation, and gene editing studies in *Vaccinium* species. (A) Chart displaying the number of published studies on regeneration, transformation, and gene editing in *Vaccinium* species from 1978 to 2025. (B) Distribution of published shoot regeneration studies among *Vaccinium* species. (C) Distribution of different explant types in *Vaccinium* regeneration studies.

Subsequent studies reported shoot regeneration across different *Vaccinium* species, mostly in *V*. *corymbosum* (47%), representing the most extensively studied species in the genus (Fig. 1B). The remaining works have been conducted in *V*. *vitis*-*idaea, V*. *angustifolium*, *V*. *macrocarpon,* and other *Vaccinium* species (Fig. 1B). Collectively, these studies highlight key factors influencing regeneration efficiency, including genotype, PGRs, explant source, and culture conditions (Table 1). To better understand the role of different factors influencing shoot regeneration, we systematically examined and discussed each factor in the following sections.

### Impact of genetic background on shoot regeneration

The regenerative capacity of plants is strongly influenced by their genetic makeup. Shoot regeneration arises from the interplay between intrinsic genetic potential and external stimuli that trigger cellular reprogramming and initiate organogenesis or embryogenesis [17]. In *Vaccinium*, the genetic background markedly affects regeneration outcomes. For instance, numerous studies in blueberry have reported that optimal plant growth regulator combinations are often genotype-specific [13, 18–29]. Some cultivars, such as ‘FL14–242,’ ‘Biloxi,’ and ‘Emerald,’ reached regeneration efficiencies of 75%–90% when subjected to as many as seven different PGR concentrations or combinations, suggesting that for certain cultivars, the regenerative response may be less constrained by genetic factors [25, 26] (Table 1). Building on these observations, a recent study proposed that selecting cultivars representing the genetic diversity of a southern highbush blueberry germplasm collection could facilitate the development of regeneration protocols applicable to genetically related cultivars, although this hypothesis remains to be experimentally validated [25].

These genotypic differences are underpinned by variation in the expression of developmental regulatory genes [30]. Transcriptomic comparisons in blueberry revealed contrasting expression between the highly regenerating cultivar ‘Blue Muffin’ and the recalcitrant ‘O’Neal’, particularly in genes linked to auxin signaling and transcription factors such as *ENHANCER OF SHOOT REGENERATION* (*VcESR*) and *WUSCHEL* (*VcWUS*) [12]. *ESR* encodes an APETALA2/Ethylene Responsive Factor (AP2/ERF) transcription factor that acts downstream of cytokinin perception to directly activate *WUS* expression, thus establishing shoot meristem identity in *Arabidopsis* [31, 32]. *WUS,* in turn, encodes a homeodomain protein that sustains stem cell fate through a feedback loop with the CLAVATA3 (CLV3) peptide signaling pathway [33–35]. This suggests that blueberry cultivars with low initial *VcESR* expression may not trigger shoot organogenesis, even when external cytokinins are applied. Functional analysis has demonstrated that *VcESR* plays a critical role in promoting shoot regeneration by modulating a broad network of endogenous cytokinin- and auxin-related genes [12]. This mechanistic understanding could explain why recalcitrant cultivars such as ‘O’Neal’ remain unresponsive across various PGR combinations.

Beyond transcriptional regulation, chromatin accessibility by epigenetic reprogramming at key developmental loci, including *WUS* and *ESR*, represents an additional layer of control [36]. This involves the removal of repressive H3K27me3 marks deposited by Polycomb Repressive Complex 2 (PRC2) at key developmental loci, thereby silencing tissue-specific gene programs and reactivating embryonic developmental networks [15, 37]. Therefore, variation in regenerative capacity likely reflects differences in cellular plasticity and epigenetic competence, together with the influence of culture conditions and environmental cues [12, 38].

Overall, these findings reinforce the conclusion that genotypic background is a major determinant of shoot regeneration in *Vaccinium*, while integrative cluster-based selection methods and gene expression–based analyses offer promising avenues to mitigate genotype dependency.

### Effect of the explant on shoot regeneration

Selecting an appropriate explant is a critical determinant of shoot regeneration success [39, 40]. In *Vaccinium*, leaves are the most commonly used explants, followed by stems. However, other tissues including callus, petioles, axillary buds, or hypocotyls are also used (Fig. 1C, Table 1). This preference likely reflects the higher morphogenic competence of leaves. While leaves generally outperform stems in shoot induction, exceptions exist depending on genotypes. For instance, in blueberry, the cultivar ‘Brigitta Blue’ exhibited the highest regeneration from leaves (88%), whereas ‘Toro’ showed the highest regeneration with stems (40%) [23]. Nonetheless, reported stem-preferred regeneration may be biased by shoot emergence from axillary buds rather than true organogenesis [20, 24].

Explant age, orientation, and physiological state strongly influence regenerative capacity in *Vaccinium*. Early studies in blueberry and lingonberry showed that younger tissues generally regenerate more frequently and vigorously than older tissues, likely reflecting greater developmental plasticity and higher totipotency [22, 41]. However, comparisons of shoot culture age have produced inconsistent results, sometimes even within the same cultivar, indicating that age alone does not reliably predict regeneration outcomes [19, 42, 43]. Culture history further modulates regeneration responses, as frequent subculturing has been shown to enhance shoot induction in several blueberry cultivars [26]. In addition, explant orientation plays a role, with higher regeneration commonly observed when the adaxial leaf surface is in contact with the culture medium, potentially due to differences in tissue polarity, stomatal distribution, or gas exchange [29, 41, 44]. Collectively, these findings highlight that regeneration efficiency reflects a complex interaction among genotype, physiological status, and culture conditions rather than a single determining factor.

### Influence of media on shoot regeneration

Basal culture media is essential for plant regeneration, providing necessary nutrients and vitamins for the newly developed shoots [45]. Different media formulations have been used for *Vaccinium* regeneration, including woody plant medium (WPM), modified WPM (MWPM), Murashige and Skoog (MS), Chu’s N6, Linsmaier and Skoog (LS), Zimmermann’s Z-2, Nitsch’s, olive medium (OM), and Knop’s media (Table 1). WPM and MWPM are commonly preferred due to their nutrient composition, which suits the specific physiological needs of woody plants, as their lower salt concentration and balanced ammonium-to-nitrate ratio closely mimic natural conditions compared to higher salt media like MS, which may induce osmotic stress and hyperhydricity [46–48].

Nonetheless, media effectiveness varies by genotype. For example, in the blueberry cultivar ‘O’Neal’, six different media were tested, and OM outperformed WPM, MS, and others in promoting adventitious shoot regeneration [49]. This enhanced performance may be due to OM’s higher nitrogen, glutamine, and phosphorus content, which meet the specific requirements of this genotype. Moreover, OM contains nitrogen in both ammonium and nitrate forms, both favorable for blueberry growth [50]. Other studies have examined media modifications to improve plant morphology and growth. For instance, MWPM increased growth and reduced reddening in the blueberry cultivar, ‘Northland’, by supplying higher phosphorus and nitrogen levels [51]. These findings highlight the importance of optimizing basal media formulations according to genotype-specific nutritional requirements, particularly the balance and availability of nitrogen and phosphorus, as well as nitrogen source, which together play a central role in maximizing shoot regeneration efficiency in *Vaccinium*.

### Influence of plant growth regulators on shoot regeneration

While basal media supply the essential nutrients for tissue culture, they alone are insufficient to induce the developmental reprogramming needed for shoot regeneration [39, 52, 53]. The type, concentration, and combination of PGRs are critical regeneration determinants in *Vaccinium*, with responses varying widely between species and cultivars (Table 1). Cytokinins are the primary drivers of shoot induction, promoting cell division, meristem activity, and organogenesis, while auxins mainly initiate dedifferentiation and pluripotency during early stages of regeneration [53–55].

At the molecular level, cytokinin is perceived by membrane-localized histidine kinase receptors that initiate a phosphorelay cascade through histidine phosphotransfer proteins to nuclear type-B arabidopsis response regulator (ARR) transcription factors, which activate *WUS* expression and promote *ESR* transcription to establish shoot meristem fate [35, 56, 57]. Type-A ARRs, which are induced by cytokinins, also reduce this signal, explaining why high cytokinin levels don’t always enhance regeneration [58, 59]. In parallel, auxin promotes dedifferentiation by triggering Transport Inhibitor Response 1/Auxin-Signaling F-Box proteins (TIR1/AFB)-mediated breakdown of Aux/IAA repressors, which frees auxin response factor (ARF) transcription factors [60, 61]. The released ARF transcription factors can then bind to auxin-responsive elements (AuxREs) to activate the transcription of downstream genes [62]. Collectively, the balance between cytokinin and auxin signaling determines whether regenerating cells commit to shoot organogenesis or revert to callus, thereby suggesting why PGR ratios optimized across different genotypes and species reflect a fundamentally conserved developmental outcome.

Both natural cytokinins (zeatin [Z], zeatin riboside [ZR], trans-zeatin [tZ], and 2-isopentenyladenine [2iP]) and synthetic cytokinins (thidiazuron [TDZ], and N-2-chloro-4-pyridyl)-N′-phenylurea [CPPU]) are widely employed for tissue culture in the genus (Table 1). Auxins such as indole-3-butyric acid (IBA), 1-naphthaleneacetic acid (NAA), and indole-3-acetic acid (IAA) are used variably, with many cultivars requiring minimal or no auxin supplementation for effective shoot regeneration [18, 63].

Comparative analyses across several *Vaccinium* species suggest that natural cytokinins tend to promote shoot regeneration more effectively than synthetic cytokinins, although the magnitude of the response varied among genotypes and species [19, 25, 64, 65]. The relatively better performance of conjugated natural cytokinins, such as ZR, likely results from their slower hydrolytic release of biologically active free-base forms into the culture medium, which allows for a more sustained and temporally controlled cytokinin signal during shoot organogenesis [19, 66]. Other studies have reported effective shoot regeneration with synthetic cytokinins in specific genotypes and species (Table 1). This could be associated with greater resistance to cytokinin oxidase (CKX)–mediated degradation in synthetic cytokinins such as CPPU and TDZ. This reduced degradation can lead to rapid accumulation and prolonged, but less regulated, receptor activation, and may be more effective in specific genotypes, especially when endogenous cytokinin metabolism limits the regeneration capacity [25, 67, 68]. Among the synthetic cytokinins, TDZ is known for its dual cytokinin- and auxin-like activity, modulating endogenous hormone signaling and enhancing both indirect organogenesis and somatic embryogenesis [44, 69].

Taken together, these observations highlight the complex, genotype-dependent hormonal regulation of shoot regeneration in *Vaccinium*, where cytokinins maintain meristem identity and drives shoot formation, and auxins modulate early developmental reprogramming. In particular, Z/ZR/tZ alone or in combination with other cytokinins such as 2iP, consistently enhanced shoot regeneration across multiple cultivars (Table 1), whereas TDZ-based treatments yielded highly variable outcomes and were ineffective in recalcitrant genotypes [25, 64]. It has also been observed that the same cultivar responded differently to identical PGRs in separate studies, possibly due to media composition, physiological stage, or environmental factors. For instance, ‘Aurora’ regenerated optimally with TDZ + NAA in MWPM [29] but required only TDZ in WPM [70]. Similar variability is observed in lingonberry cultivar ‘Erntedank’, with optimal regeneration achieved using either TDZ or Z, depending on the study [71, 72]. These findings suggest that multiple PGR treatments can support effective shoot regeneration, although their success could be influenced by specific environmental and physiological conditions.

### Effect of environmental and physiological factors on shoot regeneration

Cellular reprogramming and organogenesis are strongly modulated by environmental conditions, particularly light, playing a central role in *in vitro* regeneration by influencing photosynthesis, anatomical development, and physiological responses [37, 73, 74]. Variations in light quality, intensity, direction, and photoperiod can substantially affect regenerative capacity and growth outcomes. In *Vaccinium*, higher photosynthetic photon flux (PPF) significantly enhances shoot regeneration and shoot number, as shown in four highbush blueberry cultivars, where a PPF of 55 μmol m^−2^ s^−1^ led to higher regeneration frequencies compared to 18 μmol m^−2^ s^−1^ under ZR treatment. The same PPF condition led to reduced regeneration under TDZ treatment in one of the genotypes, implying genotype and cytokinin dependence on regenerative capacity [19].

Pretreatment of explants is another key strategy to enhance regeneration efficiency. Dark pretreatment, for instance, has been shown to increase shoot numbers in blueberry, lingonberry, lowbush blueberry, and bilberry [19, 29, 41, 75, 76]. This effect is likely due to preservation of endogenous growth regulators, increased tissue dedifferentiation, and reduced accumulation of inhibitory compounds such as oxidative polyphenols in etiolated tissues [77, 78].

In summary, shoot regeneration in *Vaccinium* species is strongly affected by genotype, explant type, PGRs, and growth conditions. Successful regeneration results from a complex interaction of these factors. Overall, the reported studies also highlight the importance of optimized, species-specific protocols to achieve efficient and reproducible *in vitro* regeneration for *Vaccinium* species.

## An overview of past and current research on *Vaccinium* transformation and gene editing

Plant transformation involves introducing a gene of interest (GOI) into plant cells, achieving stable genomic integration, and subsequently regenerating whole plants. In *Vaccinium*, transformation efforts have predominantly relied on biological delivery methods via *Agrobacterium*-mediated transformation (AMT) (Figs 2A–C and 3A). Commonly used *Agrobacterium* strains include EHA105, LBA4404, GV3101, C58C1, and AGL0, in both stable and transient transformation studies (Table 2). Among these, EHA105 is the most frequently employed strain. However, comparative evaluations of its efficiency relative to other strains are limited, with only a small number of studies directly addressing this question [29, 43]. Consequently, systematic assessments of *Agrobacterium* strain performance across different *Vaccinium* species and cultivars are needed to identify more efficient transformation strategies for the genus.

**Figure 2:**
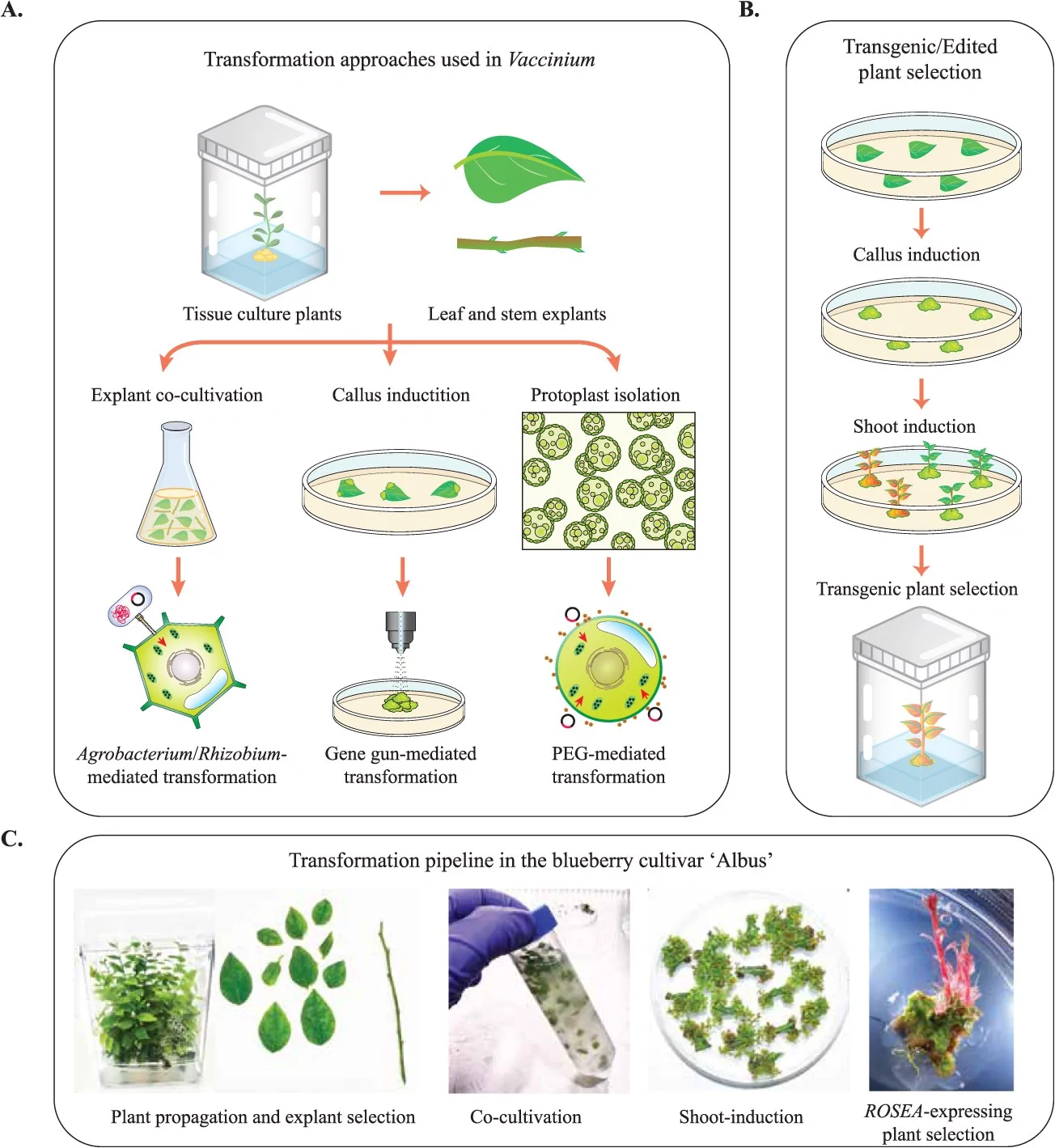
Schematic overview of genetic transformation and genome editing workflows in *Vaccinium*. (A) The process begins with the excision of explants from *in vitro*-maintained donor plants used as a source of explants, most commonly leaf or stem segments. Gene delivery into explant tissues can be achieved using either biological or physical methods. The left panel illustrates Agrobacterium-mediated transformation using *A. tumefaciens* or *R. rhizogenes*. The central panel depicts biolistic transformation, in which gold particles coated with plasmid DNA are delivered into callus tissue using a gene gun. The right panel shows protoplast-based transformation, where polyethylene glycol (PEG, represented by red dots outside the cell membrane) facilitates plasmid DNA uptake (represented by red and black circles). (B) Following transformation, explants are transferred to regeneration media containing selective agents to promote callus induction and selection for transformed tissues. Shoot formation typically occurs within 2 months, after which regenerated transgenic or edited shoots (shown in orange) are isolated and transferred to individual culture vessels for further propagation and evaluation. (C) Example of the transformation workflow applied to the blueberry cultivar ‘Albus’, resulting in ‘*ROSEA*’-expressing transgenic event (indicated by the distinct pigmentation pattern in the transformed tissue), as reported by Karn et al. [25] at the University of Florida Blueberry Breeding and Genomics Program.

**Figure 3:**
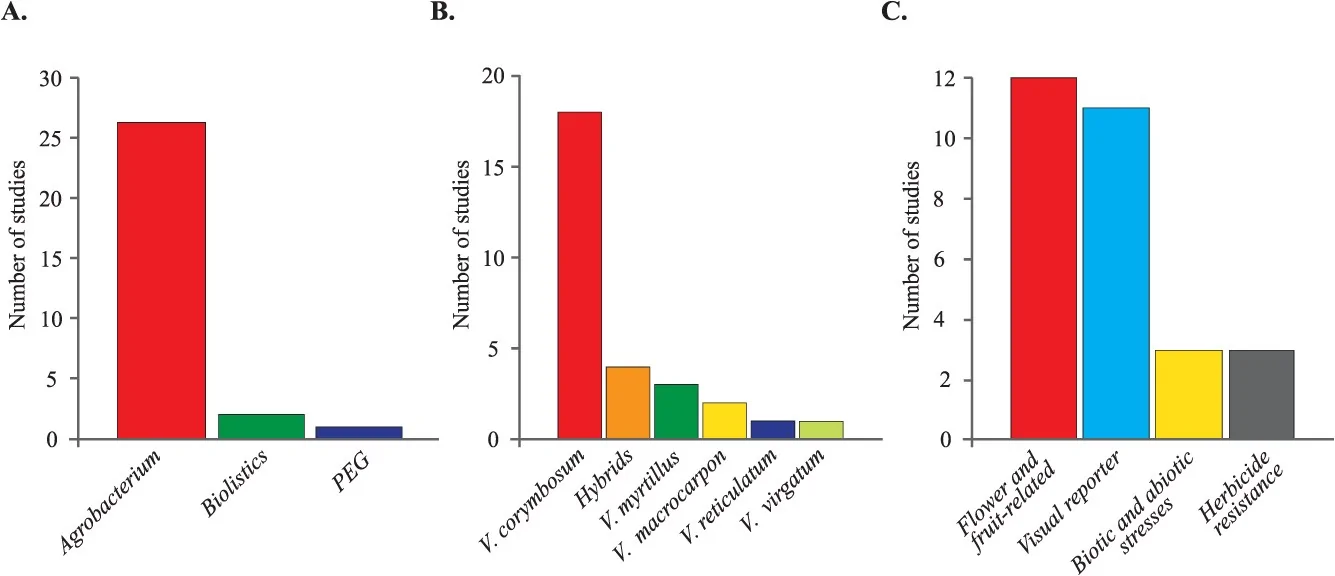
Trends in transformation studies in *Vaccinium* species. (A) Number of publications by gene-delivery method: *Agrobacterium*-mediated transformation is most frequent, whereas particle bombardment and PEG-mediated approaches are less common. (B) Distribution of published transformation studies across *Vaccinium* species, showing a predominance of blueberry (*V. corymbosum*). (C) Reported target traits from both transient and stable transformation studies in the genus.

Nonbiological gene delivery methods, including particle bombardment and polyethylene glycol (PEG)–mediated transformation, have been applied less frequently in *Vaccinium* (Figs 2B and 3A, Table 2), but may represent valuable alternatives for species or cultivars that are recalcitrant to AMT, as demonstrated in other polyploid plant systems including sugarcane, alfalfa, and potato [79–81]. However, successful implementation of these approaches in *Vaccinium* depends on the availability of tissues amenable to direct DNA delivery, particularly callus-based regeneration systems. To date, adventitious buds have been used for particle bombardment–mediated gene delivery in cranberry, resulting in limited success in recovering the expected phenotypes [82]. In blueberry, nonembryogenic callus has been employed for protoplast isolation, but plant regeneration from these protoplasts has not yet been reported [83]. This suggests that the choice of explant for particle bombardment and protoplast-based transformation is critical for achieving efficient regeneration and transformation. In this context, the use of embryogenic callus systems, coupled with optimized regeneration protocols, could improve transformation efficiency, reduce chimerism, and enable more effective nonbiological transformation approaches in *Vaccinium*.

Overall, most transformation studies in *Vaccinium* have focused on *V. corymbosum* and its hybrids, although successful reports also exist for other species, including *V. macrocarpon*, *V. myrtillus,* and *V. reticulatum* (Fig. 3B). The predominance of blueberry reflects the limited availability of well-established and reproducible transformation protocols across the genus. As observed for regeneration, transformation response may be genotype dependent [84], underscoring the need to expand research efforts within the genus. Developing robust transformation systems for additional *Vaccinium* species, together with strategies to improve transformation efficiency, will be essential to enable downstream applications such as genome editing.

### Advancements in *Vaccinium* transformation

The first attempt to evaluate the ability of *A. tumefaciens* to infect *Vaccinium* was conducted in the early 1990s, demonstrating that wild-type strains T37, C58, and A281 could induce gall formation in young hybrid seedlings (*V. corymbosum* × *V. elliottii*) [85]. This pioneering work was succeeded by the first stable transformations in blueberry and cranberry using *Agrobacterium*- and particle bombardment–mediated methods, respectively, delivering the *β-glucuronidase* (*GUS*), *neomycin phosphotransferase II* (*NPTII*), and *Bacillus thuringiensis* crystal protein (*Bt*) genes as reporter and selection markers [82, 86]. Building on these foundations, subsequent research has focused on optimizing key transformation parameters to improve transformation efficiency across *Vaccinium* species and genotypes.

In AMT, success depends not only on strain selection but also on infection and co-cultivation conditions, including bacterial density, acetosyringone concentration, co-cultivation duration, and selectable markers system employed. The optimal combination of these parameters is often species and genotype specific. Studies in *Vaccinium* have reported the highest transformation efficiency with bacterial inoculum density ranging from OD_600_ = 0.5 to 0.8, while densities higher than this were shown to increase explant necrosis without improving transformation efficiency [29, 87, 88]. Acetosyringone is critical for efficient AMT in *Vaccinium*, as its absence has been associated with reduced transformation efficiency, likely because of insufficient activation of the *Agrobacterium vir* gene regulon required for T-DNA transfer. Accordingly, 100 μM acetosyringone during inoculation and co-cultivation has become a widely used condition in *Vaccinium* transformation protocols [14, 29, 43, 89].

Co-cultivation duration is another critical factor; several studies indicate effective transformation occurs within 4–6 days, and deviations from this significantly reduce transformation success [88, 89]. Beyond these parameters, recovery of stable transformants further depends on the choice of plant selectable antibiotic. Kanamycin (*NPTII*), phosphinothricin (*Bar*), and hygromycin (*hptII*) are the most widely employed across *Vaccinium* transformation studies (Table 2), though selection stringency and marker efficacy vary considerably among genotypes and require optimization before transformation [14, 25, 90, 91]. The following sections summarize advancements in *Vaccinium* transformation that are critical for the refinement and application of NBTs in this genus and are organized by targeted traits (Fig. 3C).

#### Visual reporter genes in advancing *Vaccinium* transformation

One of the most common approaches for assessing transformation efficiency is the use of visual marker genes. In *Vaccinium*, this strategy has been widely implemented, focusing on three main visual reporter systems: (i) intronized *GUS*, (ii) green fluorescent protein (*GFP*)/enhanced *GFP* (*eGFP*), and (iii) *RUBY* (encoding enzymes for betalain biosynthesis pathway) (Table 2). Early studies established *GUS* as a reliable proof-of-concept reporter for stable transformation in several blueberry cultivars and cranberry, although its dependence on substrate staining and tissue destruction limits its broader application [29, 43, 86]. Subsequent studies adopted *GFP* for nondestructive, real-time visualization of transformed tissues. Both stable and transient *GFP* expression have been reported in *V. reticulatum, V. corymbosum, V. virgatum, V. myrtillus*, and several hybrids (Table 2) [14, 89, 92], including the most recent application *via* PEG-mediated transformation in protoplast [83].

**Table 2:** Reported transformation studies in *Vaccinium*

Approach	Species	Genotype	Explant	Delivery system (Strain)	**Gene**	Selection	Improved/studied trait	Validation system	Transformation efficiency (%)	Reference
*Stable transformation*	*V*. *corymbosum* (Highbush blueberry)	‘Albus’	Leaf	*A*. *tumefaciens* (EHA105)	Green Fluorescent protein (GFP) and Rosea	25 mg/L Kan	Proof of concept	PCR and GFP expression	2%	[25]
			Leaf	*R. rhizogenes* (Ar. A4)	RUBY	NS	Proof of concept	PCR and *RUBY* expression	53.7%	[94]
		‘Aurora’	Leaf	*A*. *tumefaciens* (EHA105)	Beta-glucuronidase (*GUS)*	10 mg/L Kan	Proof of concept	GUS expression and PCR	15.3%	[29]
					Flowering locus T (*VcFT*)	10 mg/L Kan	Early flowering	PCR and RT-PCR	NA	[186]
					Suppressor Constans 1 (*VcSOC1K*)	50 mg/L Kan	Flowering	Phenotypic, RT-PCR, and southern blot	4.4%	[99]
		‘Berkeley’	Leaf	*A*. *tumefaciens* (EHA105 and LBA4404)	*GUS*	50 mg/L Kan	Proof of concept	GUS expression	NA	[43]
				*A*. *tumefaciens* (LBA4404, C58 and AGL0)	*GUS*	2 mg/L Gen	Proof of concept	GUS expression	NA	[187]
		‘Bluecrop’	Leaf	*A*. *tumefaciens* (EHA105 and LBA4404	*GUS*	50 mg/L Kan	Proof of concept	GUS expression	NA	[43]
				*A*. *tumefaciens* (EHA105)	*GUS*	10 mg/L Kan	Proof of concept	GUS expression and PCR	5%	[29]
		‘Blue Muffin’ (BM)	Leaf	*A*. *tumefaciens* (EHA105)	*ENHANCER of SHOOT REGENERATION* (*VcESR*)	NA	Shoot regeneration	PCR	NA	[12]
		‘Brigitta’	Leaf	*A*. *tumefaciens* (EHA105)	intron-interrupted *GUS*	10 mg/L Kan	Proof of concept	GUS expression and PCR	10%	[29]
		‘Colossus’	Leaf	*R. rhizogenes* (Ar. A4)	RUBY	NS	Proof of concept	PCR and *RUBY* expression	85.2%	[94]
		‘Duke’	Leaf	*A*. *tumefaciens* (EHA105 and LBA4404)	*GUS*	50 mg/L Kan	Proof of concept	GUS expression	NA	[43]
		‘Elliott’	Leaf	*A*. *tumefaciens* (EHA105 and LBA4404)	*GUS*	50 mg/L Kan	Proof of concept	GUS expression	NA	[43]
		‘Georgiagem’	Leaf	*A*. *tumefaciens* (EHA105 and LBA4404)	*GUS*	50 mg/L Kan	Proof of concept	GUS expression	NA	[43]
		‘Jersey’	Leaf	*A*. *tumefaciens* (EHA105 and LBA4404)	*GUS*	50 mg/L Kan	Proof of concept	GUS expression	NA	[43]
		‘Legacy’	Leaf	*A*. *tumefaciens* (EHA105)	*GUS*	10 mg/L Kan	Proof of concept	GUS expression and PCR	5%.	[29]
					Bialaphos resistance (*BAR*)	0.1 and 2 mg/L GSA	Herbicide resistant	PCR, and southern blot	7%	[97]
					*BAR*	0.1 mg/L GSA	Herbicide resistant	Phenotypic, PCR, and southern blot	1.5% to 7.9%	[91]
					C-repeat bind- ing factor (*VcCBF*, renamed later as *VcDDF1*)	10 mg/L Kan	Cold tolerance	PCR, southern blot, GUS assay, Freezing tolerance in vitro, qRT PCR	6%	[188]
					Basic region/leucine zipper (*VcbZIP55)*	15 and 30 mg/L Kan	Anthocyanin synthesis	qRT PCR	NA	[189]
					*Ruby1* Egfp	10 mg/L Kan	Proof of concept	PCR, GFP expression, qRT PCR, and anthocyanin content analysis	6.5%	[88]
		‘Optimus’	Leaf	*R. rhizogenes* (Ar. A4)	RUBY	NS	Proof of concept	PCR and *RUBY* expression	43.7%	[94]
		‘Reveille’	Leaf	*A*. *tumefaciens* (EHA105 and LBA4404)	*GUS*	50 mg/L Kan	Proof of concept	GUS expression	NA	[43]
		‘Sharper’	Leaf	*R. rhizogenes* (Ar. A4)	RUBY	NS	Proof of concept	PCR and *RUBY* expression	29.6%	[94]
		‘Sierra’	Leaf	*A*. *tumefaciens* (EHA105 and LBA4404)	*GUS*	50 mg/L Kan	Proof of concept	GUS expression	NA	[43]
		‘Sunrise’	Leaf	*A*. *tumefaciens* (C58C1)	*NPTII*	10 mg/L Kan	Proof of concept	PCR	NA	[165]
				*A*. *tumefaciens* (EHA105 and LBA4404)	*GUS*	50 mg/L Kan	Proof of concept	GUS expression	NA	[43]
		‘Toro’	Leaf	*A*. *tumefaciens* (EHA105 and LBA4404)	*GUS*	50 mg/L Kan	Proof of concept	GUS expression	NA	[43]
	*V. elliottii* (Elliott’s berry)	‘Elliottii’	Leaf	*R. rhizogenes* (Ar. A4)	RUBY	NS	Proof of concept	PCR and *RUBY* expression	1.9%	[94]
	*V. reticulatum* (ohelo berry or Hawaiian blueberry)	‘Red Button’	Leaf	*A*. *tumefaciens* (GV3101)	*GFP*	100 mg/L Kan	Proof of concept	PCR and visual inspection	12.82%	[14]
	*V. macrocarpon* (canberry)	‘Pilgrim’	Axillary buds	Particle bombardment	*BAR*	0.25 mg/L GSA and Kan^a^	Tolerance to herbicide	Herbicide resistance assay	NA	[98]
		‘Stevens’	Axillary buds	Particle bombardment	*GUS* and Bacillus thuringiensis (*Bt*)	300 mg/L Kan	Enhance insect resistance	GUS expression, PCR, Southern blot, and insect feeding bioassay	34%	[82]
					*BAR*	0.25 mg/L GSA and Kan^a^	Tolerance to herbicide	Herbicide resistance assay, Southern blot	NA	[98]
	*V*. *myrtillus* (Billberry)	NA	Leaf and stem	*A*. *tumefaciens* (GV3101, EH105 and LBA4404), sonication and Agro-infiltration	*Ruby*, *GUS* and *VmMYBA1*	10 and 20 mg/L Hyg	Visual marker and anthocyanin synthesis	GUS expression, reddish or magenta color expression, PCR, and gene overexpression	4.45%	[87]
	*V. staminium* (Deerberry)	‘AP3’	Leaf	*R. rhizogenes* (Ar. A4)	*RUBY*	NS	Proof of concept	PCR and *RUBY* expression	5.8%	[94]
	*V*. *corymbosum* x (*V*. *corymbosum* x *V*. *elliottii*)	Genotype -NA	Seedling and stem	*Agrobacterium* strains: T37, C58, A281, A518, and B6.	Natural transfer of DNA from non-disarmed strains	NA	Proof of concept	Crown gall formation	7.1% (T37) 7.1% (C58) 12.5% (A281) 0 (A518) 0 (B6)	[85]
	(*V*. *corymbosum* x *V. angustifolium*) (Hybrid)	‘North Country’	Leaf and stem	*A. tumefaciens* (LBA4404)	Intron-containing *GUS*	No antibiotic selection	Proof of concept	PCR and gene overexpression	3.9%	[86]
		‘Northland’	Leaf	*A*. *tumefaciens* (EHA105)	Late embryogenesis abundant (*TaLEA*)	30 and 40 mg/L Kan	Cold tolerance	PCR, RT-PCR, and Southern blot	NA	[51]
					Chalcone synthetase (*CHS*) and *GFP*	50 mg/L Hyg	Visual marker generation	*GFP* and *CHS* expression	49.02%	[89]
	*V*. *corymbosum* NHB hybrid (‘Draper’ x ‘Legacy’)	NA	Leaf	*A*. *tumefaciens* (GV3101)	*VcMYBA1*	NA	Anthocyanin content	Gene overexpression	NA	[101]
*Transient transformation*	*V*. *corymbosum* (Highbush blueberry)	‘Aurora’	Leaf	*A*. *tumefaciens* (EHA105)	*GUS*	NA	Visual marker	GUS expression	15.3%	[29]
		‘Blue crop’	Leaf	*A*. *tumefaciens* (EHA105)	*GUS*	NA	Visual marker	GUS expression	5.6%	[29]
		‘Blue muffin’	Fruit	*A*. *tumefaciens* (EHA105)	*eGFP*	NA	Visual marker	GFP expression	79%	[92]
		‘Biloxi’	Fruit	*A*. *tumefaciens* (EHA105)	*eGFP*	NA	Visual marker	GFP expression	67%	[92]
		‘Chandler’	Fruit	*A*. *tumefaciens* (EHA105)	*eGFP*	NA	Visual marker	GFP expression	80%	[92]
		‘Duke’	Fruit	*A*. *tumefaciens* (GV3101)	*VcMYB1*	NA	Anthocyanin content	Gene overexpression	NA	[190]
		‘Eureka’	Fruit	*A*. *tumefaciens* (GV3101)	Anthocyanidin synthase (*VcANS*) and *VcMYBA1*	NA	Anthocyanin content	VIGS	NA	[102]
		‘F3L’	Leaf	*A*. *tumefaciens* (GV3101)	Chalcone synthase (*VcCHS*)	NA	Anthocyanin content	Flavonoid and anthocyanin content analysis	NA	[103]
		‘Legacy’	Fruit	*A*. *tumefaciens* (EHA105)	*eGFP*	NA	Visual marker	GFP expression	70%	[92]
		‘Legacy’	Leaf and fruit	*A*. *tumefaciens* (EHA105)	Basic region/leucine zipper (*VcbZIP55)*	NA	Anthocyanin content	Increased anthocyanin expression, qRT-PCR, and anthocyanin content analysis	NA	[189]
		“O’Neal’	Fruit	*A*. *tumefaciens* (EHA105)	*eGFP*	NA	Visual marker	GFP expression	87% (mature fruit) 13% (immature fruit)	[92]
		‘Summit’	Fruit	*A*. *tumefaciens* (EHA105)	*eGFP*	NA	Visual marker	GFP expression	70%	[92]
	*V*. *virgatum* (Rabbiteye blueberry)	‘Homebell’	Fruit	*A*. *tumefaciens* (EHA105)	*eGFP*	NA	Visual marker	GFP expression	3%	[92]
		‘Powderblue’	Fruit	*A*. *tumefaciens* (EHA105)	*eGFP*	NA	Visual marker	GFP expression	38%	[92]
		‘Tifblue’	Fruit	*A*. *tumefaciens* (EHA105)	*eGFP*	NA	Visual marker	GFP expression	25%	[92]
								GFP expression		
	*V*. *myrtillus* (Billberry)	NA	Fruit	*A*. *tumefaciens* (EHA105)	*eGFP*	NA	Visual marker generation	GFP expression	25%	[92]
		NA	Fruit	*A*. *tumefaciens* (GV3101)	*VmMYBPA1.1*	NA	Anthocyanin synthesis	qRT-PCR, VIGS, and flavonoid content analysis	NA	[191]
	(*V*. *corymbosum* X *V. angustifolium*) (Hybrid)	‘Northland’	Protoplast	Polyethylene glycol (PEG)-mediated	GFP, Na+--preferential transporter (*VcHKT1*) and calcineurin B-like protein (*AtCBL1*)	NA	Visual marker and proof of concept	GFP expression	40.40%	[83]
	*V. virgatum* (Rabbiteye hybrid derivative)	‘Pink lemonade’	Fruit	*A*. *tumefaciens* (EHA105)	*eGFP*	NA	Visual marker	GFP expression	18%	[92]

Abbreviations: NA, not available; NS, no selection; Kan, kanamycin; Gen, geneticin; GSA, glufosinate ammonium; Hyg, hygromycin; Bia, bialaphos; VIGS, virus-induced gene silencing.

a Kanamycin concentration is not reported.

An emerging and effective alternative is the use of the *RUBY* reporter gene [93]. This visual marker produces stable red pigmentation, eliminating the need for fluorescence or substrate-based assays. *RUBY* has been successfully used in *V. myrtillus* transformation [87] using *A. tumefaciens* and across different *Vaccinium* species via *Rhizobium rhizogenes* [94]. More recently, the *ROSEA* reporter gene was used in the blueberry cultivar ‘Albus’ [25], expanding the repertoire of visual markers available for *Vaccinium* transformation (Fig. 2C). However, the reliability of color-based reporters may vary depending on cultivar background and growth conditions, as pigmentation can overlap with endogenous coloration, potentially complicating the identification of true transformants. Similarly, autofluorescence can further influence plant screening when GFP or any of its variants are used [95]. Collectively, these visual marker systems have provided the foundation for optimizing transformation parameters such as co-cultivation duration, bacterial strain, explant age, and genotype across *Vaccinium* species.

#### Biotic and abiotic stress resistance in *Vaccinium*

Research focused on improving biotic and abiotic stress resistance in *Vaccinium* remains limited, largely due to the historical lack of efficient and genotype-independent transformation systems (Fig. 3C; Table 2). Early proof-of-concept was provided by the introduction of the *Bt* gene into cranberry cultivar ‘Stevens’, which conferred resistance to lepidopteran pests [82], but no subsequent efforts have targeted resistance to major pests or diseases despite increasing biotic pressures in *Vaccinium* crops. In contrast, abiotic stress tolerance, particularly cold resistance, has been more extensively explored through overexpression of the *Dwarf and Delayed Flowering* (*VcDDF1*) gene, resulting in enhanced freezing tolerance and activation of cold-responsive and reactive oxygen species (ROS)-related pathways. Notably, *VcDDF1* transgenics improved freezing tolerance without detrimental effects on plant architecture [96].

Herbicide resistance represents another trait of interest in *Vaccinium* (Fig. 3C, Table 2), where expression of the *bialaphos resistance (Bar*) gene conferred glufosinate tolerance in blueberry cultivar ‘Legacy’ and cranberry cultivar ‘Pilgrim’, with stable and heritable resistance confirmed in greenhouse evaluations [97, 98]. Overall, the scarcity of studies in these areas reflects technical constraints in transformation efficiency and regeneration, along with the long juvenile period, which have historically limited the application of biotechnology tools for stress-resilience traits in *Vaccinium*.

#### Flower and fruit quality–related traits

Understanding the metabolic pathways underlying flowering and fruit quality–related traits has become a major research focus in *Vaccinium* species (Fig. 3C, Table 2). Overexpressing the MADS-box gene *Suppressor of Overexpression of Constans 1 K* (*VcSOC1K*) in the blueberry cultivar ‘Aurora’ increased yield-related components by modulating flowering time [99]. This K-domain transcription factor activates multiple flowering pathways, promotes early flowering, enhances bud formation, and collectively increases yield potential.

*Vaccinium* crops are also distinguished by their high accumulation of phenolic compounds with strong antioxidant activity, making them valuable sources of health-promoting metabolites [100]. Among these, anthocyanins represent the predominant class, with concentrations among the highest reported in food crops [100]. Efforts to identify genes and transcription factors involved in the anthocyanin biosynthetic pathway have been successful using virus-induced gene silencing (VIGS). This approach enabled effective downregulation of the *VcANS* gene in blueberry and the transcription factors *VcMYBA1*, *VcMYBPA1.1*, and *VmMYBPA2.2 in bilberry* [101, 102]. In contrast, functional characterization by overexpression of *chalcone synthase* (*VcCHS*) in the ‘F3L’ and ‘Northland’ cultivars enhanced anthocyanin accumulation by upregulating multiple flavonoid biosynthetic genes, confirming its role in the flavonoid pathway [89, 103].

Apart from the above-mentioned traits, transformation has also been used to overcome regeneration constraints, a major limitation in *Vaccinium* genetic engineering [12]. Overexpressing *VcESR*, an AP2/ERF transcription factor that stimulates cytokinin- and auxin-related pathways, has produced phytohormone-free shoot regeneration in transgenic lines [12]. These advances collectively demonstrate the maturation of transformation systems in *Vaccinium*. Both stable and transient approaches have facilitated improvements in stress tolerance, herbicide resistance, fruit quality, and yield-related traits, while advancing gene function studies (Table 2). Despite substantial progress, transformation efficiency remains highly dependent on genotype and explant type, which continues to be a bottleneck for broader application across the genus.

However, no *Vaccinium* cultivars developed through transgenic approaches have been commercially released. This limitation may partly reflect the difficulty of generating stable transgenic events with agronomically or commercially relevant traits, as much of the existing research has focused on protocol optimization or the use of reporter genes. In addition, technical constraints, extended breeding timelines, and persistent public and regulatory concerns surrounding genetically modified perennial crops have further slowed progress. Genome editing represents a promising alternative, as it enables precise genetic improvement without the stable integration of foreign DNA, thereby alleviating regulatory and consumer acceptance barriers. Parallel efforts in regulatory frameworks, public awareness, and stakeholder engagement will be essential to support the successful adoption and deployment of these technologies in *Vaccinium* breeding programs.

### Integrating gene editing tools for accelerated *Vaccinium* breeding progress

Recent advances in NBTs have enabled the precise modification of DNA sequences in plant cells through genome editing [104]. These approaches have been successfully applied to several crops, complementing conventional breeding programs and providing a faster and more precise alternative for trait improvement [105]. Unlike transgenic approaches, genome-edited cultivars are not regulated as genetically modified organisms (GMOs) in several countries, thereby reducing regulatory barriers for field testing and commercialization [106]. However, the application of CRISPR-based systems in *Vaccinium* remains relatively recent (Fig. 1A), with only a limited number of successful cases reported, primarily in highbush blueberry (Table 2).

The first reported gene editing study in *Vaccinium* was conducted in two highbush blueberry cultivars, ‘Blue Muffin’ and “O’Neal’, targeting the *CENTRORADIALIS* (*VcCEN*) gene, a negative regulator of flowering, using CRISPR/Cas9 [84]. Although the expected early-flowering phenotype was not initially observed, subsequent work successfully achieved the desired trait [107], establishing the foundation for targeted mutagenesis in *Vaccinium*. Later, a knockout experiment using both Cas9 and Cas12a, targeted a GUS-expressing transgenic ‘Aurora’ cultivar, yielding low editing efficiency (5.5%) with Cas9 and no detectable edits with Cas12a [90]. A subsequent CRISPR/Cas9 study targeting the phytoene desaturase (*VcPDS*) gene, an essential enzyme in the carotenoid biosynthetic pathway, produced albino phenotypes [108]. However, similar to earlier work, editing efficiency remained low, likely due to the polyploid nature of blueberry, which challenges simultaneous modification of all homologous gene copies [109]. Notably, most of these early studies did not achieve complete tetra-allelic deletions, emphasizing the need for improved strategies to enhance genome-editing efficiency and mutation detection in this genus. This limited performance of first-generation CRISPR systems highlights the need to broaden the genome-editing toolkit for *Vaccinium*; importantly, the lack of editing observed with early Cas12a versions should not be taken as a fundamental limitation of alternative CRISPR systems in *Vaccinium*.

Furthermore, to address the low editing efficiencies observed in *Vaccinium*, it is essential to increase the intracellular abundance of functional gRNA–Cas9 complexes, with promoter optimization among the most effective strategies. Promoter activity often varies across species due to differences in transcriptional regulation, chromatin context, and RNA processing. In blueberry, comparisons between CRISPR/Cas9 constructs driven by heterologous promoters (AtU6 for gRNA and CaMV 35S for Cas9) and those using *Vaccinium*-specific promoters (VcU6 for gRNA and VcUBQ3b for Cas9) demonstrated that native promoters substantially increased both gRNA and Cas9 expression [92]. Furthermore, to address the low editing efficiencies observed in *Vaccinium* is essential to increasing the intracellular abundance of functional gRNA–Cas9 complexes, with promoter optimization among the most effective strategies. Promoter activity often varies across species due to differences in transcriptional regulation, chromatin context, and RNA processing. In blueberry, comparisons between CRISPR/Cas9 constructs driven by heterologous promoters (AtU6 for gRNA and CaMV 35S for Cas9) and those using *Vaccinium*-specific promoters (VcU6 for gRNA and VcUBQ3b for Cas9) demonstrated that native promoters substantially increased both gRNA and Cas9 expression [92]. Correspondingly, genome-editing efficiency was markedly enhanced, reaching up to 100% mutated allele frequency in some edited lines [107]. Collectively, these results highlight promoter optimization as an important strategy for improving CRISPR-based genome editing in *Vaccinium* crops. Alternative CRISPR systems and delivery strategies that may further improve editing efficiency and broaden applicability across *Vaccinium* species are discussed in the Future Strategies for Vaccinium Improvement through NBTs section.

## Challenges associated with the application of biotechnological tools in *Vaccinium*

The successful application of NBTs in *Vaccinium* species faces substantial constraints that limit their commercial implementation. These challenges come from both inherent biological characteristics of the genus and technical limitations in current methodologies.

Several economically important species, including highbush, lowbush, and rabbiteye blueberries, as well as small cranberry, are tetraploid to hexaploid and exhibit high heterozygosity [3]. This genomic complexity hinders precise editing, as all homologous gene copies must be modified simultaneously to produce a complete mutant phenotype [108]. To date, only four blueberry genome-editing studies have been reported, and just one achieved tetra-allelic mutations in some edited lines (Table 3). However, editing efficiency remains low, highlighting the need for optimized editing systems capable of efficiently targeting multiple alleles while minimizing off-target effects.

**Table 3:** Reported gene editing studies in *Vaccinium*

Species	Genotype	Explant	Delivery system (strain)	**Gene editing system**	**Gene**	Selection	Improved/studied trait	Validation system	Editing efficiency (%)	Reference
*V*. *corymbosum* (Highbush blueberry)	‘Aurora’	Leaf	*A*. *tumefaciens* (EHA105)	Cas9, Cas12a	*GUS*-*A*	10 and 20 mg/L Hyg	Proof of concept	NGS, amplicon and/or	5.5% (Cas 9) 0% (Cas12a)	[90]
	‘Berkeley’	Leaf	*A*. *tumefaciens* (EHA105)	Cas9	*Phytoene desaturase* (*VcPDS*)	50 mg/L Kan	Proof of concept	Sanger sequencing	2.1% to 23.8%	[108]
	‘Blue Muffin’ “O’Neal’	Axillary bud	*A*. *tumefaciens* (EHA105)	Cas9	*CENTRORADIALIS* (*VcCEN*)	10 and 20 mg/L Kan	Early flowering	Sanger sequencing	15% and 17%	[84]
	‘Blue Muffin’	Leaf	*A*. *tumefaciens* (EHA105)	Cas9	*VcCEN*	15 and 30 mg/L Kan	Early flowering	Sanger sequencing	NA	[107]

Abbreviations: NA, not available; Kan, kanamycin; Hyg, hygromycin.

The application of NBTs in *Vaccinium* is heavily constrained by low regeneration and transformation efficiencies. Regeneration typically proceeds through indirect organogenesis via callus formation [110], a multicellular process that inherently increases the risk of chimerism where transformed and nontransformed sectors coexist within the same plant [111–113]. These chimeric structures, which include unstable mericlinal or sectorial forms [113], lead to inconsistent trait expression and necessitate multiple propagation cycles to isolate uniform lines [112, 114]. Although this could potentially be reduced through adventitious regeneration from chimeric tissues, as reported in apple and pear [115], or through protoplast-based regeneration, neither approach has been established in *Vaccinium*. Furthermore, *Vaccinium* species exhibit strong recalcitrance to AMT, with success rates ranging from 5% to 34% depending on the genotype [12]. This genotype-dependent variability is compounded by suboptimal selection stringency, which allows nontransformed cells to persist through cross-protection from adjacent modified tissues or residual *Agrobacterium* activity [111]. Consequently, achieving genetically stable, nonchimeric transgenic lines remains a significant bottleneck for the scalability of NBTs in this genus [25, 90].

Finally, the prolonged juvenile phase of *Vaccinium* species further delays both trait evaluation and breeding progress [3]. This limitation is particularly problematic for fruit-related traits and for producing homozygous mutants or evaluating segregating populations [16]. The development of new *Vaccinium* cultivars typically requires 10–20 years, with the lengthy juvenile period representing a major contributor to this extended timeline [116, 117].

## Future strategies for *Vaccinium* improvement through new breeding techniques

Advancing *Vaccinium* improvement through NBTs requires overcoming the biological and technical barriers discussed previously. Future strategies should focus on enhancing transformation and gene editing efficiency by leveraging emerging molecular tools specifically tailored to *Vaccinium* species. A key priority is the development of transgene-free edited plants to ensure genomic stability and facilitate their commercial adoption [118]. Additionally, future approaches should aim to accelerate functional genomics research and enable precise integration of desirable traits into elite cultivars. Overall, integrating these innovations within a comprehensive, omics-driven framework will be essential for the sustainable genetic improvement of *Vaccinium* crops.

### Morphogenic regulators and peptides to improve regeneration and transformation

One of the major bottlenecks to transformation and genome editing in *Vaccinium* is the genotype dependence of shoot regeneration, which limits the reproducibility and broader application of existing protocols across cultivars and species. In this context, morphogenic regulators represent a particularly promising strategy because they can stimulate developmental reprogramming in otherwise recalcitrant tissues, thereby increasing transformation competence independently of the endogenous regenerative capacity of a given genotype. Classical regulators such as *BABY BOOM* (*BBM*) and *WUSCHEL* (*WUS*) can strongly enhance regeneration [119], but their constitutive expression often induce pleiotropic effects, including abnormal growth and developmental defects.

More recently, alternative regulator combinations, including *PLETHORA 1*–*WUSCHEL-related homeobox* 5 (*PLT1*-*WOX5*) and *WOUND INDUCED DEDIFFERENTIATION 1*–*ENHANCER OF SHOOT REGENERATION 1* (*WIND1*-*ESR1*) complexes, have been shown to promote regeneration across multiple crop species while mitigating adverse developmental phenotypes, thereby overcoming key regeneration bottlenecks in recalcitrant genotypes [120, 121]. Additionally, the GRF-GIF chimeric morphogenic regulator, which combines *GROWTH-REGULATING FACTOR 4* (*GRF4*) and its cofactor *GRF-INTERACTING FACTOR 1 (GIF1)*, has notably improved transformation efficiency and speed in wheat, triticale, and rice [122]. This system reduces the need for cytokinin in shoot induction, showing its broad applicability across both monocot and dicot plant species [122–124]. In *Vaccinium*, the *WIND1*-*ESR1* complex was recently used to develop shoots directly using *R. rhizogenes–*mediated transformation, suggesting a promising application of this morphogenic regulator complex [94]. This could improve genotype-flexible transformation and editing by improving regeneration without altering plant phenotype. Although these morphogenic regulators have shown considerable potential for improving regeneration in recalcitrant crops, prolonged or constitutive activity can disrupt normal plant development. Therefore, their application depends on precise control of their expression and persistence in the plant system.

Several delivery and expression strategies can provide controlled morphogenic activity, including transient, inducible, or excisable expression systems. Transient systems enable short-term morphogenic activity, achieved through *Agrobacterium*-mediated delivery of nonintegrating T-DNA vectors, viral vectors such as geminiviruses or Tobacco Mosaic Virus–based systems, or biolistic delivery of DNA, RNA, or ribonucleoprotein complexes [125, 126]. These approaches provide sufficient morphogenic stimulus to trigger totipotency while avoiding permanent transgene integration. Inducible systems allow temporal regulation of morphogenic gene expression through chemical inducers such as β-estradiol or dexamethasone, or through heat-shock promoters [127–129]. This enables activation during tissue culture and stops upon removal of the stimulus, allowing normal plant development to resume. In contrast, excisable systems allow temporary integration of morphogenic genes during transformation, followed by their targeted removal (e.g. via *Cre*/*loxP* or *FLP*/*FRT*), resulting in normal phenotypes [126, 130]. These strategies enable the controlled use of morphogenic regulators in recalcitrant crops while preventing developmental abnormalities caused by constant overexpression.

In addition, transcriptomic profiling, regulatory network, single-nucleus RNA sequencing (snRNA-seq), and chromatin analyses, as demonstrated in wheat, have enabled the identification of central transcriptional regulators associated with regeneration competence [131], providing a framework to discover novel candidate genes for improving regeneration in *Vaccinium* species. Together, the integration of morphogenic regulators, developmental regulators, and systems-level transcriptomic approaches offers a promising path toward overcoming long-standing regeneration and transformation barriers in *Vaccinium*.

Small peptides also hold promise for enhancing regeneration and transformation efficiency in *Vaccinium*. For instance, *Regeneration Factor 1* (*REF1*), a plant elicitor peptide identified in tomato, acts as a local wound signal that promotes cell reprogramming [132]. Overexpression of *SlPEP* (the tomato *REF1* precursor) or exogenous application of synthetic *REF1* peptides to induction media significantly improved regeneration and transformation rates in tomato, soybean, wheat, and maize [132]. Such approaches could be adapted to *Vaccinium* to increase regeneration efficiency.

### Delivery-based techniques to improve transformation efficiency in *Vaccinium*

Advances in *Agrobacterium*-based systems offer new opportunities to overcome transformation barriers in *Vaccinium*. Integrating type III secretion systems (T3SS) into *A. tumefaciens* enables direct delivery of defense-suppressing effectors such as AvrPto and HopAO1, which mitigate host immune responses and enhance transformation efficiency [133]. This approach has improved transformation rates in wheat, alfalfa, switchgrass, and *Arabidopsis*, and holds potential for both transient and stable *Vaccinium* transformation [133].

Hairy root transformation mediated by *R. rhizogenes* represents another promising alternative, offering rapid generation of transgenic tissues for functional studies, recombinant protein production, and metabolite analysis [134]. In *Vaccinium*, a recent study across diverse genetic backgrounds identified *R. rhizogenes* strain Ar. A4 as particularly efficient, with transformation efficiency reaching 85.4% in ‘Colossus.’ This approach could accelerate gene validation and facilitate preliminary screening of guide RNAs before genome editing experiments. Moreover, the direct induction of shoots using developmental regulators provides a potential regeneration route that bypasses the callus phase, which may be advantageous for recalcitrant genotypes [94]. However, this strategy may also increase the likelihood of chimeric regenerants, emphasizing the importance of molecular confirmation and careful assessment of regenerated plants.

Other innovations include the ternary vector system, which combines a T-DNA binary vector with a virulence gene helper plasmid, significantly enhancing transformation efficiency in *Arabidopsis*, maize and sorghum [135, 136]. Additionally, auxotrophic *Agrobacterium* strains can improve transformation by minimizing bacterial overgrowth and tissue necrosis while maintaining effective T-DNA delivery [137]. Together, these advances form a versatile toolkit for achieving more efficient, stable, and scalable transformation in *Vaccinium*, facilitating both functional analysis and crop improvement.

### Transgene-free systems for regulatory and commercial acceptance

In perennial woody species such as *Vaccinium*, conventional approaches based on crossing and backcrossing to segregate out T-DNAs are generally impractical because of long generation cycles, high heterozygosity, and the need to preserve elite clonal genotypes. As a result, alternative strategies, including DNA-free delivery systems and graft-mediated movement of editing components [118, 138], can be implemented. These approaches may provide viable routes for generating edited plants without stable integration of foreign DNA while maintaining cultivar identity. Among these alternatives, protoplast-based delivery of CRISPR ribonucleoproteins (RNPs) has shown promising results in recalcitrant species such as raspberry [138] and grapevine [139], yielding efficient, DNA-free edited plants, supporting its potential value for other woody species. In blueberry, a protoplast isolation and PEG-mediated transient transformation protocol has been recently published [83], providing an important first step towards the development of T-DNA-free editing systems. However, regeneration of whole plants from transformed protoplasts has not yet been achieved. Because of these challenges, editing protoplasts with RNPs is currently a middle- to long-term strategic goal rather than a tool that is ready for immediate use in breeding.

Alternatively, grafting-mediated genome editing offers another route to transgene-free plants, where editing components move from transgenic rootstocks to nontransgenic scions. This system has been successfully demonstrated in *Arabidopsis* and *Brassica* [118] and could be explored for perennial fruit crops like blueberry and cranberry. Taken together, these emerging strategies could expand the toolbox for developing improved *Vaccinium* cultivars while aligning more closely with market and regulatory expectations.

Regulatory considerations further increase the value of transgene-free editing strategies for commercialization. In several major markets, including the USA, Australia, Japan, and parts of Latin America, genome-edited crops lacking stable integration of exogenous DNA may be excluded from conventional GMO regulation or evaluated through a distinct regulatory pathway, depending on the jurisdiction [140, 141]. These frameworks may reduce barriers to product development and market entry compared with transgenic approaches. In contrast, the European Union has historically applied a stricter regulatory framework, although this area remains under active policy revision [140, 142]. Accordingly, future commercialization of edited *Vaccinium* cultivars should be planned with a target market and an applicable regulatory framework in mind.

### Expanding the genome-editing toolbox

Despite the rapid growth of CRISPR-Cas platforms, which have proven highly useful in polyploid and woody crops, the *Vaccinium* genome-editing toolkit remains mostly limited to conventional Cas9 systems, highlighting a clear need for broader exploration. Among these, Protospacer Adjacent Motif (PAM)–relaxed Cas9 variants such as SpG and SpRY extend target accessibility beyond the traditional NGG motif, by recognizing NGN and near-all PAM sequences. Furthermore, the use of Cas-SF01 that recognizes ATN PAMs (where A and T represent adenine and thymine, respectively, and N represents any nucleotide) could offer substantial potential for broader guide selection due to the typically AT-rich genomes in plants [143, 144]. These advancements have enabled flexible, multiallelic editing in plants such as Arabidopsis, rice, and pepper [145, 146] and could potentially help to generate multiallelic deletions in complex polyploid crops including *Vaccinium*.

Engineered Cas12a systems have shown potential in woody perennials; LbCas12a achieved multiplex targeted mutagenesis in grapevine cells, with heat treatment enhancing editing efficiency [147], while AsCas12a Ultra and LbCas12a delivered as RNPs achieved near-complete editing in citrus protoplasts [148, 149]. This collectively indicates that Cas12a is a viable platform for editing woody crops when appropriately optimized for temperature sensitivity and delivery method. For traits requiring intervention at transcript level, such as conferring resistance to RNA viruses, Cas13 offers an effective alternative, allowing for precise RNA sequence interference without altering the underlying DNA [150].

For precise modification of alleles, base and prime editors show promise by allowing targeted nucleotide changes and small insertions without depending solely on double-strand break repair [149]. Base editing has been successfully demonstrated in citrus using adenine base editors [151] and in tetraploid potato protoplasts using cytidine base editors [152, 153]. Additionally, homology-directed repair (HDR), especially replicon-assisted HDR, offers a pathway for targeted sequence replacement in elite germplasm, though their efficiency in woody perennials is still limited and needs further refinement [154]. While precise and targeted editing remains the central goal of genome editing system, efficient cargo delivery is still a major limitation for viral delivery platforms, which can be used to develop T-DNA-free crops. Miniature and hypercompact Cas like IscB-*Ωrna* and casφ have demonstrated effective delivery in rice [155, 156]. These systems may be especially valuable for *Vaccinium*, where removing the T-DNA through outcrossing would be particularly time consuming.

Alternative nucleases, such as TnpB-like systems and Tandem Interspaced Guide RNA-Targeting Systems (TIGR-Tas), have recently expanded the range of programmable editors under development. These compact RNA-guided systems may broaden access to genomic regions that are difficult to target with conventional Cas proteins, either through distinct PAM-like targeting requirements or, in the case of TIGR-Tas, through dual-guide recognition that reduces dependence on canonical PAM sequences [157, 158]. To date, however, only TnpB has been implemented in plants. In rice and Arabidopsis, TnpB-based editors have enabled targeted mutagenesis, with reported editing efficiencies exceeding 30% [159, 160] Because of their small size and distinct targeting requirements, these nucleases may help expand the editable space of plant genomes. Their compact architecture also makes them attractive candidates for virus-induced genome editing and other delivery platforms in which cargo capacity is limited. Nevertheless, these systems are still at an early stage of development and will require optimization before they can be applied in *Vaccinium*.

Overall, these emerging platforms offer a much broader and more precise set of options than those currently used in *Vaccinium* research. Systematic comparison across species, genotypes, delivery systems, and target traits will be necessary to determine which editors are most suitable for practical application in this genus.

### Leveraging genome-wide association studies and artificial intelligence to optimize new breeding techniques

The genotype dependence of AMT in *Vaccinium* underscores the need for forward-genetic approaches to dissect the genetic basis of transformation efficiency. Genome-wide association studies (GWASs) provide a robust framework for identifying loci and alleles associated with regeneration capacity, transformation efficiency, and *Agrobacterium* response [161]. For example, a hydroxyproline-rich glycoprotein that suppresses AMT in maize was identified through GWASs [162]. Similar analyses in *Vaccinium*, where GWASs have already been applied to dissect complex traits [163], could reveal novel genetic determinants of transformation success.

Beyond genetics, AMT efficiency is influenced by multiple interacting variables such as co-cultivation time, infection duration, and bacterial strain, making it a multivariate and nonlinear process. Artificial intelligence (AI) algorithms, including artificial neural networks (ANNs), have shown promise for modeling and optimizing complex *in vitro* processes such as shoot proliferation, somatic embryogenesis, and AMT. Applying these computational approaches to *Vaccinium* may facilitate predictive modeling of transformation parameters, guiding the optimization of genotype-specific NBT protocols.

## Conclusion

Recent advances in regeneration, genetic transformation, and genome editing have created new opportunities to accelerate the genetic improvement of *Vaccinium* crops. NBTs offer clear potential to overcome the limitations of conventional breeding, which is often constrained by long juvenile phases, high heterozygosity, and polyploid genomes. Despite this progress, the broader implementation of NBTs in *Vaccinium* remains hindered by strong genotype-dependent responses, recalcitrant regeneration, and low transformation and editing efficiencies in many cultivars. Addressing these challenges will require the integration of emerging strategies, including data-driven and AI-assisted optimization of regeneration protocols, the implementation of alternative gene delivery systems such as *R. rhizogenes* and virus-based platforms, and the use of regulatory elements or expression modulators to enhance Cas protein activity. In parallel, approaches such as developmental regulator–mediated regeneration and *in planta* editing strategies may further expand the applicability of genome editing in this genus. Ultimately, sustained efforts to refine these technologies and to develop efficient, transgene-free edited *Vaccinium* cultivars will be essential for fully realizing the potential of NBTs in both basic research and breeding applications. The strategies discussed in this review have shown success in similar crop systems, and their targeted adaptation to *Vaccinium* species represents a promising path toward accelerating genetic improvement across this economically important genus.

## Data Availability

The authors have nothing to report.
